# Impact of SARS-CoV-2 subvariants on postoperative outcomes in geriatric hip fracture patients – a multinational multicentre study

**DOI:** 10.1186/s12877-025-06840-6

**Published:** 2025-12-20

**Authors:** Gregor Toporowski, Christian M. Mueller-Mai, Katherine Rascher, Jonas Wiedemann

**Affiliations:** 1 Department of General Orthopaedics and Tumour Orthopaedics, Muenster University Hospital, Albert-Schweitzer-Campus 1, Muenster, 48149 Germany; 2 Department of Trauma and Orthopaedic Surgery, St. Marien Hospital, Academic Hospital of the University of Muenster, Luenen, Germany; 3 AUC - Academy for Trauma Surgery (AUC), Munich, Germany

**Keywords:** Hip fracture, Proximal femur fracture, Orthogeriatric management, COVID-19, SARS-CoV-2

## Abstract

**Background:**

Hip fractures (HF) are among the most prevalent diagnoses in geriatric traumatology, with persistently high incidence even during the COVID-19 pandemic. Concomitant SARS-CoV-2 infection adds clinical complexity and has been associated with increased mortality and prolonged hospitalisation. This study aimed to assess the impact of SARS-CoV-2 subvariants B.1.1.7 (Alpha), B.1.617.2 (Delta), and B.1.1.529 (Omicron) on postoperative outcomes in patients undergoing surgical treatment for HF.

**Methods:**

A retrospective multicentre study was conducted using data from the German Registry for Geriatric Trauma (ATR-DGU^®^) between March 2021 and April 2022 across 119 hospitals. 12,707 patients undergoing HF surgery were included and stratified by predominant subvariant periods: Alpha (*n* = 3714), Delta (*n* = 5434), and Omicron (*n* = 3559). Each cohort was further stratified by SARS-CoV-2 status at admission.

**Results:**

During the Alpha period, in-hospital mortality and length of stay were similar between COVID-19-comorbid (8.3%, 13 days) and SARS-CoV-2-negative patients (5.4%, 15 days). In the Delta and Omicron periods, mortality was significantly higher among COVID-19-comorbid patients (14.3% and 13.9%) compared to SARS-CoV-2-negative patients (5.8%, *p* = 0.017; 5.7%, *p* < 0.001), with longer hospitalisations (17 vs. 15 days, *p* < 0.05). COVID-19-comorbid patients were more frequently institutionalised and exhibited lower levels of pre-fracture mobility compared to SARS-CoV-2-negative patients.

**Conclusion:**

COVID-19-positive patients showed lower mortality during the Alpha period and higher mortality during the Delta and Omicron periods. Overall, COVID-19 comorbidity was associated with increased in-hospital mortality and longer hospitalisation. These observations may be influenced by unmeasured confounding.

**Trial registration:**

Not applicable. This retrospective study used anonymized data from the German Registry for Geriatric Trauma (ATR-DGU^®^) and was approved by the ethics committee of the University of Muenster (reference number: 2022-268-f-S). This study was conducted in accordance with the Declaration of Helsinki.

## Introduction

Hip fractures (HF) are among the most common diseases in geriatric traumatology and represent a challenge for healthcare management [1]. Due to the shift towards an older and multimorbid population, increasing incidences of HF have been reported in European countries [2]. More than 150,000 patients in Germany experience an HF annually; approximately 85% are 70 years or older [3]. The high prevalence of comorbidities in these patients adds complexity to their clinical management and increases the demands on inpatient care [4].

The COVID-19 (Coronavirus Disease 2019) pandemic has disproportionately affected the health of geriatric patients. Accordingly, comorbidity with COVID-19 combined with HF is more likely to result in a severe outcome [5, 6]. Although the incidence of HF temporarily declined during the COVID-19 pandemic, it remained one of geriatric patients’ most frequent admission diagnoses [7]. Patients frequently presented to the emergency department with an already high COVID-19 disease burden, which severely deteriorated the surgical management conditions due to the significantly limited surgical capability [7]. In some cases, stabilising the COVID-19 condition was prioritised before HF could be surgically addressed. However, despite recent studies showing no effect of delayed surgical treatment on postoperative outcomes during the COVID-19 pandemic [8], COVID-19-comorbid patients with HF are associated with significantly higher mortality and hospitalisation time [5, 9].

COVID-19, particularly its current SARS-CoV-2 (Severe Acute Respiratory Syndrome Coronavirus 2) subvariant “Omicron,” remains a significant public health concern. Despite reduced intrinsic virulence compared to earlier strains, the heightened transmissibility of recent subvariants continues to drive substantial overall mortality, particularly among geriatric patients who are at an elevated risk of severe outcomes, hospitalisation, and death [10, 11]. The coexistence of COVID-19 and HF presents a complex interplay of medical and logistical challenges, exacerbating clinical outcomes and placing considerable strain on healthcare systems, particularly in the management of frail, multimorbid patients.

Different subvariants emerged in Germany and across the globe throughout the COVID-19 pandemic, which varied in the pathogenesis of COVID-19 [6]. One of the first subvariants of concern was B.1.1.7 (Alpha), which was predominant in Germany starting from December 2020 up to summer 2021 [12]. The even more contagious subvariant B.1.617.2 (Delta) was detected and the prevailing subvariant until December 2021 [13]. As of January, the B.1.1.529 (Omicron) subvariant was predominant in Germany.

The aim of this study was to evaluate whether distinct SARS-CoV-2 subvariants were associated with variation in postoperative outcomes in geriatric patients undergoing HF surgery. Key clinical endpoints included in-hospital and 120-day mortality, complication rates, and hospital length of stay. While overall clinical attention to COVID-19 has declined, Omicron subvariants continue to circulate, and the susceptibility of elderly patients with multiple comorbidities remains unchanged. The findings may inform ongoing perioperative risk assessment and orthogeriatric care strategies under conditions of continued viral presence.

## Patients and methods

### Study design

The study period was set from March 2021 to April 2022. According to official genomic surveillance data from the Robert Koch Institute, this period was subdivided into the following periods: Alpha (B.1.1.7) became the dominant variant in calendar week 8/2021 (>50% of all sequenced infections), Delta (B.1.617.2) in calendar week 26/2021, and Omicron (BA.1) in calendar week 51/2021. The present study therefore includes the Alpha period from March to June 2021 (period 1), the Delta period from July to December 2021 (period 2), and the Omicron period (BA.1/BA.2) from January to April 2022 (period 3), before the emergence of BA.4/BA.5 from calendar week 17/2022 [12–14].

Prior to March 2021, the wild-type strain was predominant in Germany [12]. This period was not included in the analysis because ATR-DGU data on SARS-CoV-2–positive HF patients from this phase have already been reported separately by Pass et al. in 2022 [15], and nationwide vaccination of the elderly population had not yet been established [16]. Therefore, only the period beginning with the defined predominance of Alpha (from calendar week 8/2021) was considered for the present study.

The observation period was deliberately limited to April 2022, as in May 2022, the clinical the emergence of BA.4/BA.5 subvariants with significantly changed clinical appearance reduced the initial Omicron subvariant (BA.1/BA.2) to < 50% [17]. Notably, the WHO formally recommended nirmatrelvir/ritonavir (Paxlovid) for high-risk patients on 22 April 2022 [18]. Consequently, data were only available including April 2022.

The Registry for Geriatric Trauma (AltersTraumaRegister DGU^®^, ATR-DGU) of the German Trauma Society (Deutsche Gesellschaft für Unfallchirurgie, DGU) was founded in 2016 to improve geriatric trauma care. The geriatric trauma centres certified by the DGU must conduct standardised assessments of the patients treated. During the observation period from March 2021 to April 2022, 119 centres from Germany, Switzerland and Austria were certified. Since July 2020, SARS-CoV-2 status has been recorded by the ATR-DGU.

Data analysed in this study were acquired prospectively from the ATR-DGU using standardised and certified data recording tools [19]. According to the criteria of the DGU, certificated geriatric trauma centres are obliged to submit pseudonymised data of their patients aged 70 years and older with HF, pathological femur fractures and peri-implant femur fractures with an indication of surgery to the ATR-DGU. The data is collected in five consecutive time phases: Admission, before surgery, first week after surgery, discharge/transfer, and optionally follow-up on day 120 after surgery. The follow-up examination includes questions about walking ability, further surgery, and the health-related quality of life questionnaire EQ-5D on days 7 and 120 after surgery.

The data were retrospectively evaluated following approval by the ATR-DGU, granted via a peer-review procedure in accordance with publication guidelines laid down by the Working Committee on Geriatric Trauma Registry of the DGU and the University of Muenster Ethics Committee (registration number: 2022-268-f-S). This study is registered under the ATR-DGU-ID 2022-004. Clinical trial number: not applicable.

Our findings are reported according to the Strengthening the Reporting of Observational Studies in Epidemiology (STROBE) guidelines [20].

### Participants

Patients were included if they met the predefined criteria of the ATR-DGU registry:

- age ≥ 70 years, as this threshold defines geriatric HF care within the registry.
- proximal femoral fracture, including femoral neck, intertrochanteric and subtrochanteric fractures (definition: classification by AO (association for osteosynthesis) and OTA (orthopaedic trauma association)).
- surgical treatment in an ATR-DGU-certified orthogeriatric trauma centre.

Patients were excluded if they had.

- periprosthetic, peri-implant or pathological fractures due to different pathophysiology and treatment algorithms.
- conservative (non-operative) treatment.
- duplicate entries.
- missing SARS-CoV-2 status or mandatory core data.

SARS-CoV-2 infection was defined as a PCR-confirmed SARS-CoV-2 test on admission or during hospital stay.

### Outcome measures

Patients in periods 1, 2, and 3 were stratified into COVID-19-comorbid and SARS-CoV-2-negative. The primary outcome was defined as mortality and duration of hospital stay. In addition, the ability to walk before fracture and on admission, the period from admission to surgical treatment, the ISAR (Identification of Seniors at Risk) score [21], the ASA (American Society of Anaesthesiologists) score, the living situation before fracture, treatment with anticoagulation, additional injuries, the walking ability on the seventh day after surgery, the living situation at discharge and reoperations were compared between the groups.

Based on these outcomes, this study addressed two predefined research questions: [1] whether postoperative outcomes, particularly in-hospital mortality, differed across the Alpha, Delta and Omicron periods, and [2] whether the association between SARS-CoV-2 comorbidity and postoperative outcomes varied across these periods.

### Statistical analysis

The statistical evaluations were performed with the statistical software R version 4.0.2. For descriptive analyses, the median and the interquartile range (IQR) were calculated for non-parametric continuous data. Median values were compared using the Wilcoxon signed-rank test and dichotomous variables with the Chi² test. Absolute and relative frequencies represented all categorical data. The results of the EQ-5D-3 L questionnaire (EuroQol five-dimension three-level questionnaire [11]) were transformed into a single value for QoL using the time trade-off algorithm validated for Germany [22]. Linear and logistic regression models were used to examine the impact of SARS-CoV-2 subvariants on outcomes after controlling for ASA score, sex, age, and type of HF, including interaction analyses where appropriate. Results are reported as regression coefficients (β) for linear regression and odds ratios (OR) for logistic regression, along with their 95% confidence interval. For regression models stratified by period, COVID-19–negative patients served as the reference category. Differences were considered statistically significant when *p* < 0.05.

## Results

### Study population

**Fig. 1:**
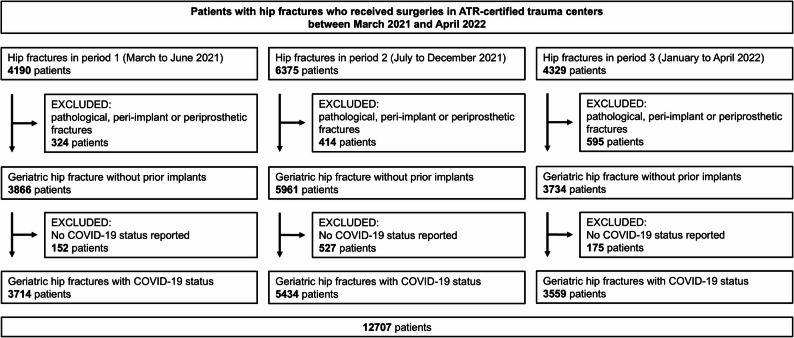
Strengthening the Reporting of Observational Studies in Epidemiology (STROBE) flow diagram for patients with hip fractures (HF) treated between March 2021 and April 2022 documented in the Registry for Geriatric Trauma (AltersTraumaRegister, ATR) of the German Trauma Society (Deutsche Gesellschaft für Unfallchirurgie, DGU)

4190 patients received surgical treatment for HF in period 1. 324 patients were excluded due to pathological, peri-implant or periprosthetic fractures. 152 patients were excluded as no SARS-CoV-2 status was reported. Thus, 3714 patients were included in our study in period (1) In period 2, 6375 patients received surgical treatment for HF. 414 patients were excluded due to pathological, peri-implant or periprosthetic fractures. 527 patients were excluded as no SARS-CoV-2 status was reported. Thus, 5434 patients were included in our study in period (2) In period 3, 4329 patients received surgical treatment for HF. 595 patients were excluded due to pathological, peri-implant or periprosthetic fractures. 175 patients were excluded as no SARS-CoV-2 status was reported. Thus, 3559 patients were included in our study in period 3 (Fig. 1).

**Fig. 2:**
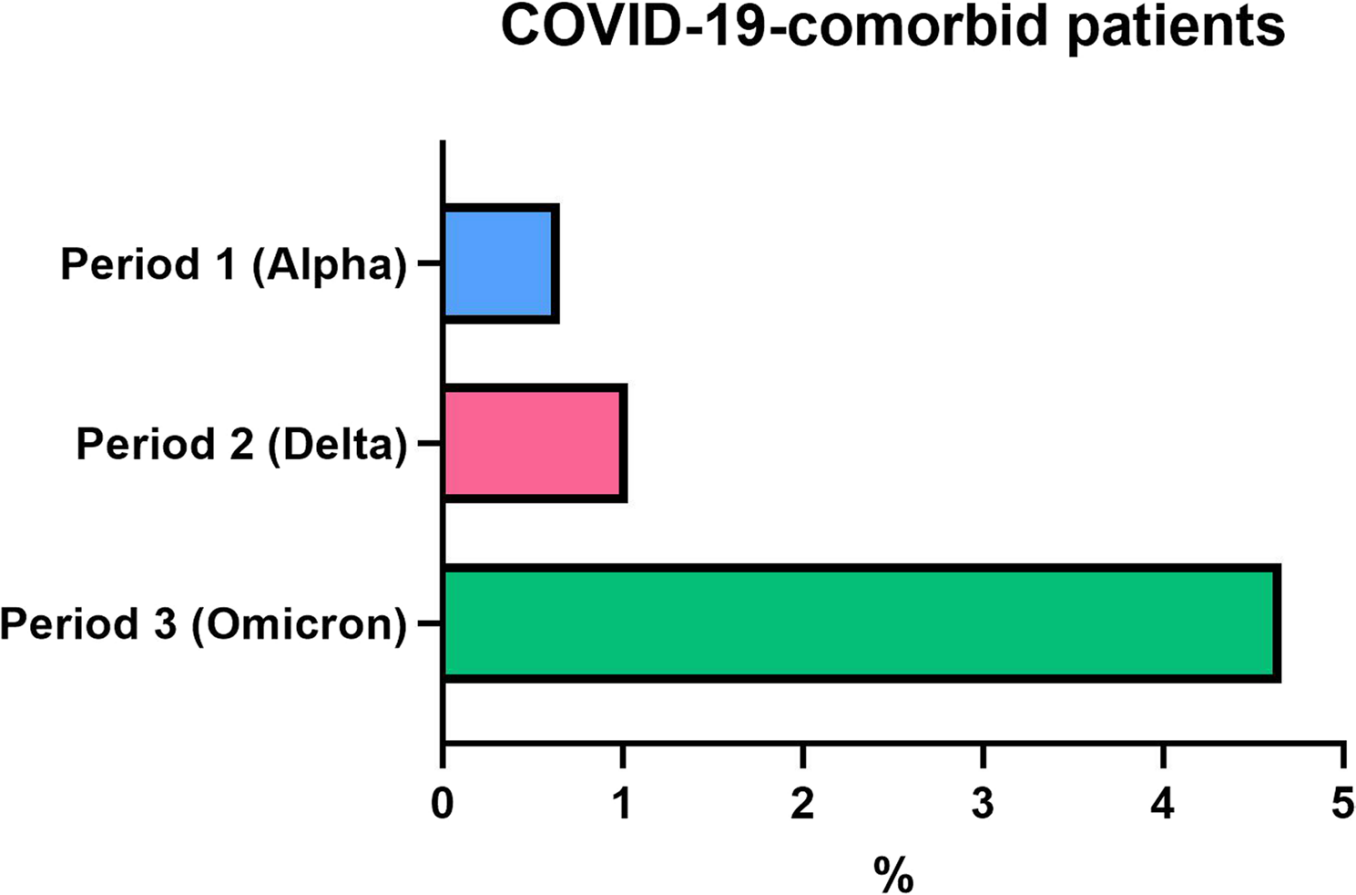
Proportion of geriatric HF patients who tested positive for SARS-CoV-2 at admission, stratified by the dominant subvariant period: Alpha, Delta, and Omicron. Values represent the percentage of total HF cases registered in the respective time periods

Of the total 3714 cases from 112 geriatric trauma centres in period 1, 24 patients were SARS-CoV-2-positive, representing a rate of 0.65%. In period 2, out of 5434 cases from 116 geriatric trauma centres, 56 patients were SARS-CoV-2-positive at admission, corresponding to a rate of 1.03%. In period 3, out of 3559 cases from 119 geriatric trauma centres, 166 patients were SARS-CoV-2-positive at admission, resulting in a rate of 4.66% (Fig. 2). Patient baseline characteristics are presented in Table 1.

**Table 1:** Patient baseline characteristics and preoperative functional status, comorbidities, and social background in SARS-CoV-2-positive and -negative geriatric HF patients across periods of Alpha, Delta, and Omicron variant predominance. Data are shown as n (%) or median (interquartile range, IQR). Comparisons were conducted using Chi-squared and Wilcoxon rank-sum tests as appropriate

Parameter		Period 1 (Alpha)			Period 2 (Delta)			Period 3 (Omicron)		
		*COVID-neg*	*COVID-pos*	*p Value*	*COVID-neg*	*COVID-pos*	*p Value*	*COVID-neg*	*COVID-pos*	*p Value*
*n*		3690	24	-	5378	56	-	3393	166	-
Age (years, IQR)		85 (9)	87 (7)	0.054	85 (9)	84 (9)	0.472	84 (9)	85 (9)	0.175
Sex (w:m %)		71:29	71:29	1	71:29	68:32	0.677	70:30	68:32	0.640
Anticoagulation therapy (%)		56	67	0.407	54	57	0.758	52	56	0.298
Additional injuries (%)		11	13	0.786	10	7	0.640	10	6	0.143
Housing situation	*Home*	2830 (78%)	14 (58%)	0.038	4190 (79%)	40 (71%)	0.358	2695 (80%)	101 (62%)	<0.001
	*Nursing Home*	760 (21%)	9 (38%)		1408 (20%)	15 (27%)		625 (19%)	61 (37%)	
	*Hospital or other*	46 (1%)	1 (4%)		73 (1%)	1 (2%)		26 (1%)	2 (1%)	
Ambulatory status	*Independent*	1228 (36%)	7 (33%)	<0.001	1835 (37%)	15 (30%)	<0.001	1169 (37%)	43 (30%)	<0.001
	*With cane*	345 (10%)	3 (14%)		470 (10%)	7 (14%)		294 (9%)	11 (8%)	
	*With crutches or walker*	1177 (35%)	7 (33%)		1699 (35%)	18 (36%)		1059 (34%)	48 (33%)	
	*Limited to indoor*	516 (15%)	4 (19%)		733 (15%)	9 (18%)		499 (16%)	37 (26%)	
	*Non-ambulatory*	109 (3%)	-		168 (3%)	1 (2%)		98 (3%)	4 (3%)	
ASA score (IQR)		3.0 (0)	3.0 (0)	0.366	3.0 (0)	3.0 (0)	0.193	3.0 (0)	3.0 (0)	0.004
ISAR score (IQR)		3.0 (2.0)	3.0 (2.0)	0.327	3.0 (2.0)	3.0 (1.0)	0.004	3.0 (2.0)	3.0 (2.0)	<0.001

### Period 1 – Alpha subvariant

In period 1, preoperative housing situation was significantly inferior in patients with HF and COVID-19 comorbidity, with a lower proportion of patients residing at home (58%) and a higher proportion of patients living in nursing homes (38%) or hospitals (4%) compared to SARS-CoV-2-negative patients (78%/21%/1%, *p* = 0.038). In contrast, no difference in preoperative walking ability was observed compared with SARS-CoV-2-negative patients with HF (*p* = 0.866). Concurrently, the in-hospital mortality rate was not significantly different for COVID-19 comorbidity (8.3%) compared to SARS-CoV-2-negative patients (5.4%, *p* = 0.851). The median hospitalisation time for SARS-CoV-2-negative patients was 15 days (IQR 12) and showed no difference from COVID-19-comorbid patients (13 days, IQR 10, *p* = 0.876). There were also no significant differences between sexes, age, ISAR score, ASA score, current anticoagulation, or additional injuries. At the 120-day follow-up, only 9 of the 22 SARS-CoV-2-positive patients treated could be contacted. Although statistical differences were identified when comparing the SARS-CoV-2-negative cohort, the small sample size limits the reliability of these findings, and no conclusive differences could be observed in terms of mortality, residential location, walking ability, or hospital readmission (Table 1).

### Period 2 – Delta subvariant

In period 2, preoperative housing situations did not differ between patients with HF and COVID-19 comorbidity and SARS-CoV-2-negative patients (*p* = 0.358), as did walking ability (*p* = 0.670). The median preoperative ISAR score was 3.0 (IQR 2.0) in COVID-19-comorbid patients, significantly higher than in SARS-CoV-2-negative patients (3.0, IQR 1.0, *p* = 0.004). The mortality rate was significantly higher with COVID-19 comorbidity (14.3%) compared to SARS-CoV-2-negative patients (5.8%, *p* = 0.017). A significant increase in median hospitalisation time was found in COVID-19-comorbid patients (17 days, IQR 16) compared to SARS-CoV-2-negative patients (14 days, IQR 11, *p* = 0.012). At 120 days after surgery, COVID-19-comorbid patients in period 2 showed a significant improvement in housing situation (17%) compared to SARS-CoV-2-negative patients (3%, *p* = 0.004) as well as an increased mortality rate of 35.0% compared to SARS-CoV-2-negative patients (11.9%, *p* = 0.004). There were no significant differences between sexes, age, ASA score, current anticoagulation, or additional injuries.

### Period 3 – Omicron subvariant

The preoperative housing situation in period 3 was inferior in patients with HF and COVID-19 comorbidity, with fewer patients residing at home (62%) and more patients residing in nursing homes (37%) compared with SARS-CoV-2-negative patients (80%/19%, *p* < 0.001). Moreover, the walking ability was significantly reduced in the COVID-19-comorbid cohort, resulting in a lower rate of independent walking (30%) and a higher rate of limited to indoor patients (26%) compared with SARS-CoV-2-negative cohort (37%/16%, *p* < 0.001). The median preoperative ASA score (3.0, IQR 2.0) and ISAR score (3.0, IQR 0) were significantly higher in COVID-19-comorbid patients compared to SARS-CoV-2-negativ patients (*p* < 0.001, *p* = 0.002). The mortality rate was significantly increased with COVID-19 comorbidity (13.9%) compared to SARS-CoV-2-negative patients (5.4%, *p* < 0.001). Moreover, the median hospitalisation time was longer in COVID-19-comorbid patients (17 days, IQR 15) compared to SARS-CoV-2-negative patients (15 days, IQR 12, *p* = 0.03). No change in housing situation (*p* = 0.774) or difference in mortality (*p* = 0.774) 120 days after surgery was observed in COVID-19-comorbid patients in period 3 compared to SARS-CoV-2-negative patients. There were no significant differences between sexes, age, current anticoagulation, or additional injuries.

The preoperative physical condition and mobility status, and the outcome parameters are detailed in Tables 1, 2 and 3, and 4.

**Table 2:** Outcome parameters of geriatric patients with (COVID-pos) and those without COVID-19 comorbidity (COVID-neg) and hip fracture (HF) across periods of Alpha, Delta, and Omicron variant predominance Group comparisons were performed using Wilcoxon rank-sum and Chi-squared tests as appropriate

Parameter	Period 1 (Alpha)			Period 2 (Delta)			Period 3 (Omicron)		
	*COVID-neg*	*COVID-pos*	*p Value*	*COVID-neg*	*COVID-pos*	*p Value*	*COVID-neg*	*COVID-pos*	*p Value*
In-house mortality (*n*)	198/3489 (5%)	2/142 (8%)	0.851	313/5056 (6%)	8/48 (14%)	0.017	181/3197 (5%)	23/142 (14%)	<0.001
Time of hospitalisation (*n*, days/IQR)	3475, 15 (12)	22, 13 (10)	0.876	5036, 14 (11)	47, 17 (16)	0.012	3172, 15 (12)	140, 17 (15)	0.030
Time to surgery (*n*, hours/IQR)	3664, 16 (16)	24, 16 (17)	0.756	5351, 16 (16)	56, 14 (16)	0.996	3325, 16 (16)	164, 17 (15)	0.104
EQ5d score 7 days after surgery (n, median/IQR)	2457, 0.701 (0.410)	19, 0.701 (0.457)	0.684	3535, 0.701 (0.410)	31, 0.378 (0.410)	0.361	2400, 0.701 (0.410)	100, 0.313 (0.638)	<0.001

**Table 3:** Multivariable analysis using logistic regression (odds ratio [OR] with 95% confidence interval [CI]) for key outcomes and linear regression (β-coefficient with 95% CI) for key predictors after surgical treatment of HF in COVID-19-comorbid patients. All models were adjusted for sex, age, ASA score and fracture type

Parameter	Period 1 (Alpha)			Period 2 (Delta)			Period 3 (Omicron)		
	*n*	* OR (95% CI)*	*p Value*	*n*	*OR (95% CI)*	*p Value*	*n*	*OR (95% CI)*	*p Value*
In-house mortality	3594	1.54 (0.24–5.49.24.49)	0.567	5239	2.85 (1.21–5.94.21.94)	0.009	3397	2.69 (1.62–4.31.62.31)	<0.001
Acute reoperation	3587	1.30 (0.07–6.32.07.32)	0.800	5138	2.67 (0.91–6.22.91.22)	0.040	138	0.69	1
Follow-up mortality	1205	1.25 (0.15–10.42.15.42)	0.838	1611	3.07 (1.10–8.62.10.62)	0.033	908	0.96 (0.35–2.62.35.62)	0.938
	*n*	*β (95% CI)*	*p Value*	*n*	*β (95% CI)*	*p Value*	*n*	*β (95% CI)*	*p Value*
Time to surgery	3648	1.02 (−8.22−10.26)	0.829	5352	−2.35 (−8.33−3.63)	0.442	3356	1.92 (−2.28−6.13)	0.370
Hospitalisation time	3462	0.75 (−2.74−4.24)	0.673	5029	5.36 (2.95–7.76.95.76)	<0.001	3177	2.43 (0.93–3.94.93.94)	0.002
EQ5d after 7 days	2427	0.01 (−0.13−0.14)	0.935	3501	−0.03 (−0.13−0.07)	0.536	2382	−0.13 (−0.18−0.07)	<0.001

**Table 4:** Follow-up outcomes at 120 days after HF surgery in SARS-CoV-2-positive and -negative geriatric patients, stratified by variant period (Alpha, Delta, Omicron). Reported outcomes include mortality, readmission, revision surgery, walking ability, and housing status. Data are shown as n (%) or median (IQR). Group comparisons were conducted using Chi-squared and Wilcoxon rank-sum tests as appropriate

Parameter		Period 1 (Alpha)			Period 2 (Delta)			Period 3 (Omicron)		
		*COVID-neg*	*COVID-pos*	*p Value*	*COVID-neg*	*COVID-pos*	*p Value*	*COVID-neg*	*COVID-pos*	*p Value*
Readmission	*No*	1152 (96%)	9 (100%)	<0.001	1455 (95%)	20 (91%)	0.615	758 (97%)	32 (100%)	<0.001
	*Yes*	44 (4%)	0 (0%)		69 (5%)	2 (9%)		25 (3%)	0 (0%)	
Walking ability	*No change*	354 (36%)	1 (14%)	0.472	425 (35%)	4 (29%)	0.660	290 (39%)	17 (57%)	0.154
	*Improved*	89 (9%)	1 (14%)		98 (8%)	2 (14%)		72 (10%)	2 (6%)	
	*reduced*	530 (55%)	5 (72%)		705 (57%)	8 (57%)		381 (51%)	11 (37%)	
Housing situation	*No change*	139 (18%)	0 (0%)	<0.001	152 (15%)	2 (17%)	0.004	86 (13%)	3 (14%)	0.774
	*Improved*	11 (1%)	0 (0%)		22 (2%)	2 (17%)		16 (2%)	1 (5%)	
	*reduced*	638 (81%)	5 (100%)		851 (83%)	8 (66%)		561 (85%)	17 (81%)	
Mortality	*Yes*	977 (87%)	7 (88%)	1	1274 (88%)	13 (65%)	0.004	778 (88%)	31 (78%)	0.774
	*No*	146 (13%)	1 (12%)		172 (12%)	7 (35%)		108 (12%)	9 (22%)	
Revision surgery	*Yes*	1124 (97%)	20 (95%)	0.619	1426 (97%)	20 (95%)	1	751 (97%)	32 (100%)	<0.001
	*No*	38 (3%)	1 (5%)		52 (3%)	1 (5%)		26 (3%)	0 (0%)	

### Multivariable analysis

During periods 2 and 3, COVID-19 comorbidity was associated with significantly increased odds of in-hospital mortality (OR 2.9, 95% CI 1.2–5.9, *p* = 0.009; OR 2.7, 95% CI 1.6–4.3, *p* < 0.001, respectively). In period 2, the odds ratio for mortality at follow-up after 120 days was 3.1 (95% CI 1.1–8.6, *p* = 0.033) (Table 3). Additionally, the duration of hospitalisation increased by 5.4 days (95% CI 3.0–7.8, *p* < 0.001) in period 2 and 2.4 days (95% CI 0.9–3.9, *p* = 0.002) in period 3. The EQ-5D index after 7 days was significantly reduced in period 3 by 0.13 points (*p* < 0.001) (Table 3).

### Comparison across periods and interaction analysis

To address the predefined research questions, we first analysed whether in-hospital mortality differed across the Alpha, Delta and Omicron periods in the overall cohort, irrespective of SARS-CoV-2 infection. In the multivariable logistic regression model adjusting for age, sex, anticoagulation therapy, additional injuries, housing situation, ambulatory status, ASA score and ISAR score, no significant differences in in-hospital mortality between periods were detected. Compared with the Alpha period, the adjusted odds ratio (OR) was 1.15 (95% CI 0.96–1.38; *p* = 0.13) for Delta and 1.12 (95% CI 0.91–1.36; *p* = 0.28) for Omicron. Mortality also did not differ significantly between Delta and Omicron (OR 1.03, 95% CI 0.86–1.24; *p* = 0.74) (Table 5).

**Table 5:** Logistic regression analysis of in-house mortality in geriatric HF patients across SARS-CoV-2 variant periods (Alpha, Delta, Omicron). Mortality risk was compared between periods in the overall cohort. Results are reported as adjusted odds ratios (OR) with 95% confidence intervals (CI) and p-values. Models were adjusted for age, sex, anticoagulation therapy, additional injuries, housing situation, ambulatory status, ASA score and ISAR score

Comparison	Adjusted OR	95% CI	*P* Value
Alpha vs. Delta	1.15	0.96–1.38.96.38	0.134
Alpha vs. Omicron	1.12	0.91–1.36.91.36	0.283
Delta vs. Omicron	1.03	0.86–1.24.86.24	0.741

Secondly, we examined whether the association between COVID-19 comorbidity and in-hospital mortality varied across variant periods. The interaction between SARS-CoV-2 status and period was statistically significant (*p* = 0.003). In the Alpha period, SARS-CoV-2 infection was not significantly associated with mortality (OR 0.26, 95% CI 0.06–1.04; *p* = 0.057), whereas it was associated during the Delta (OR 2.99, 95% CI 1.39–6.46; *p* = 0.005) and Omicron periods (OR 3.34, 95% CI 2.08–5.37; *p* < 0.001) (Table 6).

**Table 6:** Association between SARS-CoV-2 infection and in-house mortality stratified by variant period (Alpha, Delta, Omicron). Logistic regression models include SARS-CoV-2 status (positive vs negative), variant period, and the COVID-19 × period interaction term. Results are presented as adjusted odds ratios (OR) with 95% confidence intervals (CI) and p-values. All models were adjusted for age, sex, anticoagulation therapy, additional injuries, housing situation, ambulatory status, ASA score and ISAR score

Period	Effect of COVID-19 (OR)	95% CI	*P* Value
Alpha	0.26	0.06–1.04.06.04	0.057
Delta	2.99	1.39–6.46.39.46	0.005
Omicron	3.34	2.08–5.37.08.37	<0.001

## Discussion

Multiple studies have demonstrated that COVID-19-comorbid patients with concurrent HF present higher mortality rates and more extended hospital stays compared to SARS-CoV-2-negative patients [9, 15, 23]. This multinational, multicentre study provides a detailed analysis of the outcomes of patients with HF with and without COVID-19 comorbidity, stratified by the predominant subvariants: Alpha (B.1.1.7), Delta (B.1.617.2), and Omicron (B.1.1.529).

### Functional status and living situation before surgery

In our study, the preoperative housing conditions during period 1 (Alpha) and the preoperative housing situation and walking situation during period 3 (Omicron) were significantly lower in HF patients with concurrent SARS-CoV-2 infection compared to SARS-CoV-2-negative HF patients at hospital admission. Pass et al. also observed poorer walking ability and decreased living situations in COVID-19-comorbid patients with HF, analysing the onset of the COVID-19 pandemic, during which the wild-type virus was predominant [15]. This disparity likely reflects the higher exposure risk and underlying frailty of nursing-home residents, who are characterised by reduced mobility and increased susceptibility to respiratory infections such as SARS-CoV-2 [24–26].

### In-Hospital and 120-Day outcomes across variant periods

During periods 2 (Delta) and 3 (Omicron), higher in-hospital mortality rates and longer hospitalisation times were observed among SARS-CoV-2-positive patients with HF compared to SARS-CoV-2-negative patients. This trend was not evident during period 1, although a numerical increase in in-hospital mortality was detected from 5.4% to 8.3%. In period 1, which represented the shortest observation period, the Alpha subvariant had a lower incidence rate than the Delta and Omicron subvariants in periods 2 and 3, primarily due to limited vaccine availability and, thus, frequent lockdowns as well as possible under-testing [27, 28]. As a result, only 24 HF patients were reported as SARS-CoV-2-positive during this period, necessitating careful interpretation of the findings due to the small sample size and statistical limitations.

Early studies focusing on the wild-type virus already confirmed increased mortality and longer hospitalisation among COVID-19-comorbid HF patients [9, 15]. Regardless of the subvariant — particularly with the currently predominant subvariant Omicron — COVID-19 comorbidity tends to be associated with an increase in in-hospital mortality rates and hospitalisation time. A SARS-CoV-2 screening remains indicated for patients with HF to assess potential risks.

In the regression analyses performed for this study, we did not observe significant differences in overall in-hospital mortality between periods in the entire cohort. When SARS-CoV-2 status was included as an interaction term, the resulting interaction was statistically significant (*p* = 0.003). This statistical signal should, however, be interpreted cautiously: the very small number of infected patients during the Alpha period leads to wide confidence intervals, thereby limiting the robustness of the interaction and preventing firm conclusions about period-specific differences in the association between SARS-CoV-2 infection and mortality.

120 days after surgical treatment, COVID-19-comorbid patients in period 2 showed a significant deterioration in housing conditions, which was not observed in periods 1 or 3. Mortality was also significantly higher 120 days after surgical treatment in COVID-19-comorbid patients in period 2. The higher incidence and morbidity of the Delta subvariant compared to the Alpha subvariant could contribute to a more complicated postoperative course in HF patients. At the same time, the clinical course of COVID-19 in individuals infected with the Omicron subvariant is considered milder than in those infected with the Delta subvariant [29]. These associations may support our findings, but it is essential to emphasise the small number of COVID-19-comorbid patients in the cohorts, which limits statistical interpretation.

Interestingly, although the Omicron subvariant has been associated with a generally milder disease course [11], our study found that COVID-19-comorbid HF patients continued to experience prolonged hospitalisation times and higher mortality rates. Notably, 90% of the German population aged over 60 years had received at least one dose of the COVID-19 vaccine by period 3 [16], thereby providing enhanced protection against severe COVID-19 outcomes. This paradox may reflect the cumulative strain on healthcare resources and the unique vulnerabilities of the geriatric population, characterised by multiple comorbidities and frailty.

### Pathophysiological mechanisms and clinical implications

The present findings raise important questions regarding possible pathophysiological mechanisms contributing to the increased perioperative risk in COVID-19-comorbid patients, particularly during periods 2 and 3. Inflammation-induced endothelial dysfunction, hypercoagulability, and myocardial involvement have been reported more frequently in Delta-subvariant infections compared to earlier variants [29, 30]. These mechanisms may have contributed to the increased in-hospital and 120-day mortality observed in our cohort during period 2.

Beyond pathophysiological differences, pandemic-related structural factors must be considered when interpreting these findings. The decline in HF incidence during the COVID-19 pandemic and the extensive reorganisation of surgical and rehabilitation services profoundly changed clinical pathways for older adults [7, 31]. Isolation measures, delayed admissions, and fluctuating staff capacities likely influenced postoperative outcomes independently of virulence. Consequently, comparing SARS-CoV-2-positive and negative patients within the same pandemic context provides a more reliable framework than pre-pandemic comparisons under fundamentally different healthcare conditions. Moreover, the degree of healthcare system strain varied across subvariant periods and may have influenced perioperative management and patient outcomes. During the Alpha and Delta periods, hospitals operated under higher systemic pressure with increased ICU occupancy and staff shortages, whereas the Omicron period was characterised by lower admission rates and less severe disease [32]. These dynamics likely contributed to differences in patient flow, surgical timing, and postoperative care.

Many health systems imposed restrictions or reorganisation of surgical services that may have affected HF surgery timing and prioritisation. Some institutions postponed elective procedures or limited theatre capacity [32], while lockdown policies led to reduced orthopaedic procedure volumes in multiple settings [33]. These procedural constraints could have introduced delays or differential access to surgery across subvariant periods, potentially influencing postoperative outcomes beyond viral and patient-related factors.

### Study limitations

As a retrospective analysis, this study is subject to inherent biases, including selection and confounding factors. While data collection through the ATR-DGU is standardised and quality-controlled, the lack of detailed clinical data — such as vital signs, the severity of COVID-19 symptoms, vaccination status, and specific causes of mortality — limits the ability to account for relevant confounders. In particular, the very small number of SARS-CoV-2–positive patients in the Alpha subgroup reduces the statistical precision of period-specific comparisons and restricts the robustness of any observed differences between variant phases. Additionally, the relatively small number of SARS-CoV-2-positive patients overall may reduce the statistical power of the analyses. Another limitation is that pre-pandemic data could not be analysed, which restricts the possibility of comparing outcomes with the pre-COVID era. Consequently, the findings reflect only the pandemic period and should be interpreted within this context. Furthermore, the focus on specialised geriatric trauma centres may restrict the generalisability of the findings to other healthcare settings.

## Conclusion

This multinational, multicentre study presents differences in outcomes of geriatric hip fracture patients across the Alpha, Delta, and Omicron periods. During the Alpha period, SARS-CoV-2–positive patients showed in-hospital mortality and hospitalisation patterns comparable to those of SARS-CoV-2–negative patients. In contrast, during the subsequent Delta and Omicron periods, COVID-19-positive patients exhibited higher in-hospital mortality and longer hospitalisation than negative patients.

Across all periods combined, COVID-19 comorbidity was associated with higher in-hospital mortality and prolonged hospitalisation, highlighting its continued clinical relevance in geriatric hip fracture care. Interpretation of the findings must also consider the retrospective study design and the absence of important clinical variables such as vaccination status and potential changes in healthcare system strain or treatment pathways over time. Nevertheless, these results may offer clinically relevant orientation for managing geriatric hip fracture patients with concurrent SARS-CoV-2 infection during shifting pandemic circumstances.

## Data Availability

The data that support the findings of this study are available from AUC but restrictions apply to the availability of these data, which were used under license for the current study, and so are not publicly available. Data are however available from the authors upon reasonable request and with permission of AUC.

## Abbreviations

AO: Association for Osteosynthesis
AUC: Academy for Trauma Surgery
HF: Hip fracture
ATR-DGU: German Registry for Geriatric Trauma
COVID-19: Coronavirus Disease 2019
SARS-CoV-2: Severe Acute Respiratory Syndrome Coronavirus 2
DGU: German Trauma Society**/**Deutsche Gesellschaft für Unfallchirurgie
STROBE: Strengthening the Reporting of Observational Studies in Epidemiology
ISAR: Identification of Seniors at Risk
ASA: American Society of Anaesthesiologists
IQR: Interquartile range
EQ-5D-3L: EuroQol five-dimension three-level questionnaire
OR: Odds ratio
OTA: Orthopaedic Trauma Association
